# Randomised feasibility trial of a teaching assistant led extracurricular physical activity intervention for 9 to 11 year olds: Action 3:30

**DOI:** 10.1186/s12966-014-0114-z

**Published:** 2014-09-11

**Authors:** Russell Jago, Simon J Sebire, Ben Davies, Lesley Wood, Mark J Edwards, Kathryn Banfield, Kenneth R Fox, Janice L Thompson, Jane E Powell, Alan A Montgomery

**Affiliations:** Centre for Exercise, Nutrition & Health Sciences, School for Policy Studies, University of Bristol, Bristol, UK; School of Sport and Exercise Sciences, University of Birmingham, Birmingham, UK; Department of Health and Social Sciences, University of the West of England, Bristol, UK; Nottingham Clinical Trials Unit, University of Nottingham, Nottingham, UK

**Keywords:** Teaching assistant, Feasibility trial, Intervention, Children, Physical activity

## Abstract

**Background:**

Extracurricular programmes could provide a mechanism to increase the physical activity (PA) of primary-school-aged children. The aim of this feasibility study was to examine whether the Action 3:30 intervention, which is delivered by teaching assistants, holds promise as a means of increasing the PA of Year 5 and 6 children.

**Methods:**

A cluster randomised feasibility trial was conducted in 20 primary schools. Ten schools received the Action 3:30 intervention and 10 schools were allocated to the control arm. The intervention was 40 one-hour sessions, delivered twice a week by teaching assistants. The proportion of participants recruited per school was calculated. Session delivery and session attendance was calculated for intervention schools. Weekday and after-school (3.30 to 8.30 pm) moderate to vigorous intensity physical (MVPA) was assessed by accelerometer at baseline (T0), during the last few weeks of the intervention (T1) and four months after the intervention had ended (T2). The costs of delivering the intervention were estimated.

**Results:**

Five intervention schools ran all 40 of the intended sessions. Of the remaining five, three ran 39, one ran 38 and one ran 29 sessions. Mean attendance was 53%. The adjusted difference in weekday MVPA at T1 was 4.3 minutes (95% CI −2.6 to 11.3). Sex-stratified analyses indicated that boys obtained 8.6 more minutes of weekday MVPA than the control group (95% CI 2.8 to 14.5) at T1 with no effect for girls (0.15 minutes, 95% CI −9.7 to 10.0). There was no evidence that participation in the programme increased MVPA once the club sessions ceased (T2). The indicative average cost of this intervention was £2,425 per school or £81 per participating child during its first year and £1,461 per school or £49 per participating child thereafter.

**Conclusions:**

The effect of the Action 3:30 intervention was comparable to previous physical activity interventions but further analysis indicated that there was a marked sex difference with a positive impact on boys and no evidence of an effect on girls. The Action 3:30 intervention holds considerable promise but more work is needed to enhance the effectiveness of the intervention, particularly for girls.

**Trial registration:**

ISRCTN58502739

## Introduction

Physical activity is associated with reduced body mass, healthier blood lipid profiles, lower blood pressure, lower insulin levels and enhanced mental well-being among children [1]. Despite the benefits of regular physical activity, many young people do not meet the current UK recommendation of an hour of moderate to vigorous intensity physical activity (MVPA) on most days of the week [2]. Physical activity levels decline throughout childhood, with the end of primary school (UK school years 5 and 6) and start of secondary school being a key transition period for physical activity [3].

A number of different methods have been used as possible approaches to increasing children’s physical activity. The majority of these have been delivered during curriculum time and focussed on changes to physical education or health programmes [4,5]. Systematic reviews have highlighted that the effect of curriculum-based physical activity programs have been limited [6,7] and therefore alternative approaches may be useful. Extra-curricular interventions, which use school facilitates (playground, gym etc.) but do not use core teaching time, provide an opportunity in which children could be more active [6]. A 2009 systematic review identified 13 papers reporting the results from 11 different after-school interventions, of which only one had included objective assessments of physical activity [8]. None of the studies were conducted in the United Kingdom [8]. A separate 2009 review highlighted weakness in the methods used to evaluate extra-curricular interventions and identified a need for more well-controlled trials [9]. Several studies have been published since the 2009 reviews, which have further shown the potential of the after-school setting [10]. For example, the pilot evaluation of the GoGirlGo after-school programme showed an increase of around 11 minutes in the MVPA of girls when attending the programme. However, the absence of a control group means that further evaluation of the intervention is needed [10]. Similarly, evaluation of a multi-component environmental intervention delivered through US YMCA programmes resulted in ten additional minutes of MVPA in the intervention group when compared with the control group [11]. The study was, however, limited by its quasi-experimental design including possible pre-existing differences between the intervention and control sites. Thus, current evidence suggests that extra-curricular physical activity programmes hold promise but there is a lack of information from robust UK evaluations.

After-school clubs are a central element of the UK Government’s Extended Schools strategy for primary schools. Many children participate in supervised programmes for additional academic support, music, creative activities, and competitive and non-competitive sports [12]. Organised after-school programmes that focus on increasing physical activity opportunities for a broad range of children could provide an effective means of engaging inactive children in physical activity. Current extra-curricular provision is dominated by external leaders such as football or netball coaches who provide clubs for a fee [13]. However, in the current financial climate more cost-effective means of delivery of these programmes, such as more efficient use of existing school staff, are required. Teaching assistants work in the classroom setting and help teachers to support children with tasks such as reading, writing, and maths in both large groups and on a one–to-one basis. The number of teaching assistants has increased greatly in the UK since 2000 and teaching assistants now comprise around a third of the workforce in UK schools [14]. There are comparable para-professional staff members in a number of other countries including Cyprus, the USA and Australia [15–17]. Teaching assistants (and similar para-professional staff in other countries), could therefore be trained to deliver after-school physical activity programmes designed to create physical activity environments different from those delivered via the traditional curriculum (e.g., physical education/sports) and thereby increase physical activity teaching capacity within the schools. Training teaching assistants in physical activity delivery would also extend their skill-set and contribute towards their continuing professional development.

Developing interventions using behavioural theories allows particular psycho-social characteristics to be targeted and evaluated as part of the process of enhancing the effectiveness of complex interventions [18]. Self-determination theory may be particularly appropriate for understanding children’s physical activity [19]. This theory focuses on motivation for behaviour and postulates that being motivated for autonomous reasons (because physical activity is fun or provides benefits such as feelings of competence or spending time with friends) leads to more positive cognitive, affective and behavioural outcomes than does being motivated by externally controlled reasons (such as feeling pressured to be active). Autonomous motivation is hypothesised to develop according to the satisfaction derived from feelings of autonomy (choice and volition in when and how to be active), competence (feeling able to master different activities) and relatedness (feeling understood and part of a supportive physical activity environment) [20]. These factors can be undermined or facilitated by the actions and interaction style of key people in the social environment (e.g., a teacher or coach). Programmes that help primary school children feel more physically competent and confident to be active, and which engender fun and being part of a supportive team, are therefore likely to optimise children’s motivation to engage with the intervention and stay involved in physical activity.

We hypothesised that teaching assistant-led after-school programmes which focussed on increasing children’s motivation and confidence in relation to physical activity could hold promise as a means of promoting physical activity among primary school children. However, before proceeding to a large cluster randomised controlled trial more information about the potential of this approach and the feasibility of the intervention including the likely cost of the intervention and the potential sample size is required [21,22]. We therefore developed the Action 3:30 intervention and evaluated it via a feasibility trial. The specific aims of the feasibility study were to:

1. Estimate the likely recruitment, attendance, and retention rates of pupils to the Action 3:30 after-school physical activity intervention.
2. Estimate the likely impact on physical activity while the club was still running and four months after contact sessions had ended.
3. Develop a reliable costing tool and assess the feasibility of obtaining programme cost data.
4. Estimate the sample size for an adequately powered evaluation of the Action 3:30 intervention.

## Methods

### Sampling and participants

Pupils from Years 5 and 6 (9 to 10 years of age) were recruited from primary schools within Bristol, Bath and North-East Somerset (BANES), and South Gloucestershire Local Education Authorities. All 189 mainstream state-funded primary schools in these areas, with the exception of 51 already participating in concurrent studies, were invited to participate. Twenty schools were selected on a first-come-first-served basis. The number of pupils recruited from each school was limited to 30 as this was considered to be the maximum number that could be accommodated in each session. Schools with fewer than 30 pupils in Years 5 and 6 combined (n = 1) were excluded. Where more than 30 pupils volunteered to participate, 30 were randomly selected to participate in the study.

Schools were randomly assigned to intervention or control arms once baseline data had been processed. Randomization was conducted by a member of a clinical trials unit with no other involvement in the study. Allocation was purposively balanced in relation to Local Education Authority membership, deprivation, school size, ratio of Year 5 to Year 6 pupils, proportion of female participants, and mean minutes of MVPA at baseline [21,23]. All measures were assessed at baseline (time 0 - T0), during the last few weeks of the intervention period (time 0 + 20 weeks - T1) and four months after the intervention ended (time 1 + 4 months - T2). (Please note that at the T2 assessment the participants who were in Year 6 at the T0 assessment had moved to secondary school). The T1 assessment was designed to provide an indication of physical activity when the clubs were still running while the T2 assessment was designed to provide information about any sustained impact in the absence of direct contact. Intervention schools received the Action 3:30 programme (see below) and control schools received £200 towards the school fund once all data had been collected. Children in both arms of the trial received a small thank-you gift at baseline (a frisbee), at first follow-up (water bottle) and a £10 gift voucher for the final data collection. The study received ethical approval from the School for Policy Studies Research Ethics Committee at the University of Bristol (ref: Action 3:30 Project) and written parental informed consent was obtained for all participants.

#### Intervention

The Action 3:30 intervention is described in detail elsewhere [21]. Briefly, however, two teaching assistants from each school received a five day training programme that focussed on delivering a physical activity programme in an after-school environment. The intervention was based on self-determination theory and the teaching assistants were trained to facilitate sessions that covered a range of physical activities that included games, pair work and individual challenges. They were also trained in how to use an autonomy-supportive style that acknowledged pupils’ feelings and preferences, conveyed a sense of choice and provided support for children’s autonomy, competence and relatedness [24]. The focus of the intervention was on promoting children’s perceptions of autonomy, belonging and competence. Amongst a range of techniques, to promote autonomy teaching assistants were encouraged to provide children with choices within the activities, such as leading warm-ups, adaptating games (e.g., rule changes group sizes, equipment) and, there were child-led sessions in which the children chose the activities. Teaching Assistants supported competence by setting progressive activities targeting quick successes balanced with providing optimal challenge and providing specific praise for attempts as well as outcomes. Relatedness was supported through empathic TA-child interactions, TAs showing interest in the children’s lives outside the intervention and encouraging team-work.

Once trained, the assistants were asked to deliver Action 3:30 clubs twice a week for 20 weeks lasting 60 minutes each. Detailed session plans were provided for all 40 sessions which the teaching assistants were asked to deliver in the prescribed order. Every two weeks the pupils were provided with an information sheet which included activity ideas, based on the content of the last four sessions, which they could practise outside the club. Intervention schools were given £200 of equipment to provide additional resources for their Action 3:30 club and they were also reimbursed for teaching assistant time to attend training and deliver the sessions. Pupils in schools allocated to the control arm provided data at T0, T1 and T2 only and no other contact was made by the research team.

### Measures

The following measures were collected at all three time points.

#### Physical activity

Physical activity was assessed using an ActiGraph accelerometer (Model GT3X+; ActiGraph LLC, FL, USA) which was set to collect data at 30Hz for a maximum of five days including a weekend day. Participants were instructed to remove the monitor for sleeping and bathing. Actigraph accelerometers have been shown to provide estimates of energy expenditure that are closely associated with laboratory-derived energy expenditure [25]. Periods of ≥60 minutes of zero values, were defined as accelerometer “non-wear” time and were removed from the analyses [26]. To maximise the study sample participants were included if they provided at least two weekdays of valid accelerometer data (a valid day was defined as the provision of at least 500 minutes of data between 6 am and 11 pm). An after-school window was also created to detect physical activity that occurred between the end of school (3:30 pm) and evening (8:30 pm). The 8:30 pm cut-off was used as previous global positioning system data collected in Bristol has shown that very little physical activity occurs outside after 8:30 pm [27]. Mean minutes of MVPA on a weekday and in the after-school period on weekdays were derived using a cut-point of ≥2296 counts per minute [28]. Mean counts per minute (CPM) per day and during the after-school period on weekdays were also determined. The CPM data were designed to provide an indication of the overall volume of physical activity in which the children engaged and thus it was hypothesised that the CPM measures would capture smaller changes in sedentary and light activity that would not be apparent from changes in minutes of MVPA.

#### Height and weight

Child height was measured using a SECA Leicester stadiometer (HAB International, Northampton). Weight was recorded using a SECA 899 digital scale (HAB International, Northampton). Body mass index (BMI = kg/m^2^) was calculated and converted to an age and sex specific standard deviation score (BMI z-score) based on 1990 UK child growth reference curves [29] using the Stata ‘zanthro’ command [30].

#### Questionnaire

Participants were asked to complete a questionnaire consisting of a number of descriptive variables including age, gender, and home postcode. The postcode was used to derive an index of multiple deprivation (IMD) score for each participant, using the English Indices of Deprivation (http://data.gov.uk/dataset/index-of-multiple-deprivation). The IMD score estimates area deprivation based on several indicators covering a range of economic, social and housing criteria, which are combined into a single deprivation score for each small area in England. The IMD variable was used as a measure of socio-economic status and was included in the final regression models as a potential confounder.

#### Process assessments

In addition to the above measures, the number of children who attended each session in each intervention school was recorded by the teaching assistants. Registers were returned to the project team and average attendance over the number of sessions was calculated for each school. The number and proportion of pupils who attended at least half of all possible sessions was derived from this information.

#### Resources

Resource use data and actual costs incurred and claimed across nine categories of resource, ranging from consultancy to intervention delivery by teaching assistants, was collected by the project team from time-sheets and expense sheets. Costs were categorised as research costs, non-recurrent development costs, one-off training costs and recurrent programme delivery costs. Actual costs incurred were used to estimate teaching assistant training and Action 3.30 delivery costs at the school level.

#### Qualitative assessments

Exit interviews were conducted with teaching assistants and key contacts at schools, and focus groups were conducted with children in intervention schools. These data were collected to identify the elements of the intervention that worked well and aspects that could be improved. Due to space constraints these data are not presented in this paper. A link to a subsequent paper will be made available on the study website (http://www.bristol.ac.uk/sps/research/researchprojectpages/action330/index.html) when published.

### Statistical analysis

The aim of this trial was to assess the feasibility of implementing an after-school club and the analyses focussed on addressing key issues of feasibility rather than estimating group differences in outcomes. The proportion of consenting pupils from Years 5 and 6 in each school was calculated, based on the numbers of pupils in these years. Descriptive statistics - including means, standard deviations, frequencies and percentages - were calculated to compare the trial arms at baseline. To be included in the analysis, participants were required to have provided valid accelerometer data at all three time points. Student t-tests were used to compare the mean BMI, and deprivation scores for participants who were and were not included in the analyses. T-tests were also used to compare whether there was any difference in the BMI z-scores, IMD or baseline levels of MVPA between participants who had two compared with three weekdays of valid accelerometer data.

Random-effects linear regression models were used to estimate between-arm differences in four separate elements of children’s physical activity (CPM and minutes of MVPA, for weekdays and during the after-school period on weekdays) at T1 and T2. These models were adjusted for the variables used to balance the randomisation, with mean school level MVPA at baseline, and school level tax credit eligibility being replaced by individual baseline activity (MVPA or counts per minute as appropriate) and individual household IMD, with robust standard errors used to take account of the cluster randomised design [31]. Results from the fully adjusted regression models were provided, along with the crude means for each arm. As children’s physical activity has been shown to differ by child sex, the interaction between each intervention and sex was formally tested. Based on some evidence of a differential effect of the intervention for boys and girls on after-school MVPA, we re-ran all models stratified by sex. Since this was a feasibility study, between-group differences in physical activity outcome are described and interpreted using point estimates and 95% confidence intervals only, and p-values are not presented.

The school associated intra-class correlation coefficient (ICC) for weekday MVPA was calculated. The potential sample size for a future trial was calculated using a range of different combinations of alpha (0.05 or 0.01) and power (80% or 90%) and the point estimate (0.06534) or upper 95% confidence limit (0.12977) of the school associated ICC for weekday MVPA. Initially, all calculations were based on a final sample size for analysis of 24 children per school which, after allowing for a 20% loss to follow-up, would require recruitment of a sample of 30 children per school. The calculations were repeated to allow for sex-specific analyses based on a sex-specific sample size for analysis of 12 children per school which would require recruiting 15 children per school. All analyses were performed in Stata version 12.0 (College Station, Texas).

## Results

**Figure 1:**
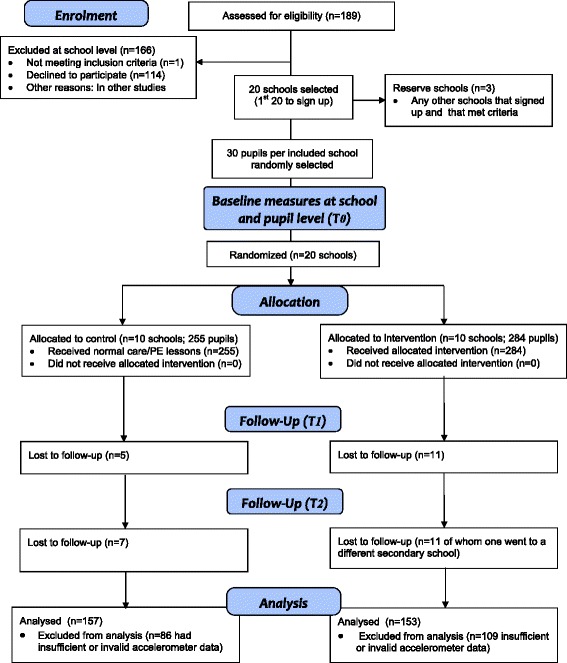
**Flow Diagram for Action 3:30 trial (based on CONSORT 2010 flow diagram).**

Recruitment, attendance, and baseline data provision rates are summarised in Tables 1 and 2. The flow of participants through the study is presented in Figure 1. A slightly higher proportion of the control group compared with the intervention group provided accelerometer data at baseline (93% vs. 88%) with all children in both groups providing questionnaire information at this time. Mean attendance over all club sessions in the 10 schools was 53% with considerable variation across the 10 intervention schools. For example, one school achieved a mean attendance of 86%with all enrolled pupils attending at least half of all sessions, whereas two schools had mean attendance of under 40% of pupils (Table 2). Five of the ten intervention schools ran all 40 of the intended sessions. Of the remaining five, three ran 39, one ran 38 and one ran 29 sessions.

**Table 1:** **Recruitment, consent rate, data provision and weekly attendance in the Action 3:30 project by school**

**School**	**Arm** ^**a**^	**N pupils in Y5 & Y6**	**Provided consent (n,%)**						**Enrolled (n,% of consenting)**		**Provided baseline accel. data (n,% of enrolled)** ^**b**^	
			**n**	**%**	**Y5**	**Y6**	**M**	**F**	**n**	**%**	**n**	**%**
1	C	47	27	57.4	9	18	16	11	27	100.0	26	96.3
3	C	104	44	42.3	20	24	18	26	30	68.2	29	96.7
5	C	107	39	36.4	21	18	23	16	30	76.9	25	83.3
7	C	38	24	63.2	16	8	12	12	24	100.0	23	95.8
11	C	52	20	38.5	7	13	8	12	20	100.0	16	80.0
13	C	96	38	39.6	21	17	18	20	30	78.9	29	96.7
14	C	119	19	16.0	10	9	8	11	19	100.0	16	84.2
15	C	164	36	22.0	17	19	22	14	30	83.3	29	96.7
17	C	52	24	46.2	14	10	7	17	24	100.0	24	100.0
20	C	48	21	43.8	11	10	9	12	21	100.0	19	90.5
All	C	827	292	35.3	146	146	141	151	255	87.3	236	92.5
2	I	91	35	38.5	17	17	12	22	30	85.7	23	76.7
4	I	55	36	65.5	17	19	11	25	30	83.3	29	96.7
6	I	109	25	22.9	11	14	11	14	25	100.0	21	84.0
8	I	96	33	34.4	22	11	12	21	30	90.9	25	83.3
9	I	105	23	21.9	11	12	14	9	23	100.0	20	87.0
10	I	120	47	39.2	27	20	23	24	30	63.8	28	93.3
12	I	111	40	36.0	23	17	20	20	30	75.0	29	96.7
16	I	92	31	33.7	13	18	9	22	30	96.8	29	96.7
18	I	66	27	40.9	13	14	15	12	27	100.0	19	70.4
19	I	115	29	25.2	13	16	12	17	29	100.0	28	96.6
All	I	960	326	34.0	167	158	139	186	284	87.1	251	88.4

^a^I = Intervention, C = Control.

^b^Numbers represent provision of valid data. (Valid = Participant’s accelerometer data met the overall wear time criteria of at least 500 minutes between 6 am and 11 pm on at least two weekdays).

**Table 2:** **Mean weekly attendance in the Action 3:30 project by school**

**School**	**N sessions**	**N pupils**		**Mean weekly attendance during intervention (based on those attending at least once)**	**N attending at least 50% of all possible sessions (based on those recruited to club)**	
		**Recruited**	**Attending at least 1 session**			
		**n**	**n**	**%**	**n**	**%**
2	40	30	27	63.3	20	66.7
4	40	30	30	85.5	30	100.0
6	39	25	19	56.0	10	40.0
8	40	30	27	48.5	15	50.0
9	38	23	21	44.5	9	39.1
10	39	30	29	39.2	9	30.0
12	39	30	29	53.9	17	56.7
16	40	30	30	36.2	8	26.7
18	40	27	23	52.8	11	40.7
19	29	29	23	45.0	11	37.9
All	384	284	258	52.8	140	49.3

In general the physical activity levels of all pupils enrolled in the control arm were similar to those in the intervention arm (Table 3). There was some evidence that pupils excluded from the final analysis due to insufficient data were slightly older and more deprived than those included in the analysis (Table 4). Further analysis indicated that there was no difference between those with valid data on either two or three weekdays for IMD (p = 0.618), BMI z-score (p = 0.412), or weekday MVPA at T0 (p = 0.271), (data not in tabular form).

**Table 3:** **Means and standard deviations (SD) for anthropometric and physical activity data by trial arm at T0**

	**Control**					**Intervention**				
	**n**	**Mean**	**SD**	**Min**	**Max**	**n**	**Mean**	**SD**	**Min**	**Max**
BMI (kg/m^2^)	255	18.4	3.3	12.8	29.7	284	18.8	3.4	13.5	33.1
BMI SDS	248	0.51	1.2	−2.6	3.3	272	0.61	1.2	−2.1	3.4
Age (yrs)	248	10.0	0.55	9.1	11.1	272	10.1	0.59	7.8	11.1
IMD	248	19.8	17.0	2.9	67.6	277	21.5	17.7	1.4	66.2
MVPA weekday (mins)	228	60.2	23.0	21.1	187.4	241	59.2	21.8	23.2	155.3
CPM weekday	228	550.2	153.9	265.3	1346.1	241	546.6	146.8	261.9	1245.9
MVPA after school (mins)*	228	13.4	8.2	1.5	71.4	241	12.1	6.7	1.6	37.4
CPM after school*	228	612.0	331.1	11.1	2074.4	241	549.4	335.3	71.4	2604.9

*MVPA recorded between 3.30 pm and 8.30 pm on schooldays (pupils with valid weekday accelerometer data).

Valid MVPA weekdays/after school: Participant’s accelerometer data met the overall wear time criteria of at least 500 minutes between 6 am and 11 pm mins on at least two weekdays.

**Table 4:** **Characteristics of participants who were included in the overall analysis compared with those who were excluded**

	**Included**			**Excluded**			**Difference**		
**Overall**	**n**	**Mean**	**SD**	**n**	**Mean**	**SD**	**Mean**	**95% CI**	**p**
BMI SDS*	310	0.58	1.2	229	0.57	1.2	−0.008	−0.21 to 0.19	0.9390
Age (yrs)	310	10.0	0.55	229	10.1	0.59	0.14	0.05 to 0.24	0.0037
IMD**	310	18.9	17.3	215	23.2	17.2	4.3	1.3 to 7.3	0.0055
Control									
BMI SDS	157	0.52	1.2	98	0.19	1.3	−0.02	−0.33 to 0.28	0.8853
Age (yrs)	157	10.0	0.53	98	10.1	0.57	0.14	0.00 to 0.28	0.0495
IMD	157	16.9	16.1	91	24.8	17.5	7.9	3.6 to 12.2	0.0004
Intervention									
BMI SDS	153	0.64	1.1	131	0.62	1.2	−0.01	−0.29 to 0.26	0.9209
Age (yrs)	153	10.0	0.57	131	10.1	0.60	0.15	0.010 to 0.28	0.0357
IMD	153	21.0	18.4	124	22.0	17.0	1.0	−3.2 to 5.2	0.6376

*BMI SDS: Age and sex standardised BMI (BMI z score).

**IMD: Index of multiple deprivation score.

Overall, the point estimates show modest benefits in favour of intervention arm at T1 but the 95% CIs are wide. The adjusted difference in weekday MVPA after participating in the programme was 4.3 minutes of MVPA (95% CI −2.6 to 11.3) (Table 5). Results of analyses stratified by sex suggest that this result was explained by an increase in MVPA amongst boys who obtained 8.6 more minutes of weekday MVPA than the control group (95% CI 2.8 to 14.5). In this group, MVPA in the after-school period increased by 4.0 minutes (95% CI 1.6 to 6.4) (Table 6). In contrast, there was no beneficial effect of participating in the club on girls’ weekday MVPA (−0.15 minutes, 95% CI −9.7 to 10.0) with a similar effect for after-school MVPA (−0.01 minutes; 95% CI −2.2 to 2.2). Again, the 95% CIs are wide. There was no evidence that participation in the programme increased MVPA once the club sessions ceased (T2) (Tables 5, 6, 7).

**Table 5:** **Physical activity data by trial arm at T0, T1, and T2 and adjusted between-group differences at T1 and T2 for pupils with at least two days of valid weekday data for T0, T1, and T2 (n = 310)**

	**Control (n = 157)**		**Intervention (n = 153)**		
	**Mean**	**SD**	**Mean**	**SD**	**I vs. C** ^**††**^ adjusted difference in means (95% CI)**
	T0				
MVPA/weekday (mins)	59.0	19.7	57.8	20.4	N/A
MVPA/weekday after school (mins)^†^	12.8	7.3	12.2	6.6	N/A
CPM/weekday	538.0	136.2	533.7	137.9	N/A
CPM/weekday after school^†^	621.3	269.9	625.5	290.2	N/A
	T1				
MVPA/weekday (mins)	65.5	21.6	65.7	27.4	4.3 [−2.6 to 11.3]
MVPA/weekday after school (mins)^†^	13.0	7.0	14.3	8.4	1.6 [0.06 to 3.1]
CPM/weekday	614.2	176.3	616.1	205.9	32.4 [−32.9 to 97.7]
CPM/weekday after school^†^	707.7	381.5	740.9	401.2	54.9 [−51.8 to 161.6]
	T2				
MVPA/weekday (mins)	55.3	22.7	54.7	21.1	0.69 [−3.4 to 4.8]
MVPA/weekday after school (mins)^†^	11.7	7.4	11.0	7.1	−0.52 [−1.7 to 0.69]
CPM/weekday	478.6	149.3	483.6	140.8	6.0 [−18.8 to 30.9]
CPM/weekday after school^†^	545.2	263.0	540.1	283.4	−5.0 [−58.5 to 48.6]

T0: Baseline; T1: T0 + 20 weeks; T2: T0 + 6 months.

**Between group differences always compare to the intervention arm and are adjusted for baseline outcome value, IMD, school size, percentage of girls recruited, percentage of Y5 pupils recruited, LEA, and school-level clustering.

^†^After school period =3.30 to 8.30 pm.

^††^I = intervention; C = control.

**Table 6:** **Physical activity data by trial arm at T0, T1, and T2 and adjusted between group differences at T1 and T2 for boys with at least two days of valid weekday data for T0, T1, and T2 (n = 126)**

	**Control (n = 157)**		**Intervention (n = 153)**		
	**Mean**	**SD**	**Mean**	**SD**	**I vs. C** ^**††**^ adjusted difference in means (95% CI)**
	T0				
MVPA/weekday (mins)	65.7	21.7	68.2	24.0	N/A
MVPA/weekday after school (mins)^†^	14.3	9.1	13.8	7.2	N/A
CPM/weekday	584.7	144.2	592.4	157.4	N/A
CPM/weekday after school^†^	671.7	316.7	681.3	307.0	N/A
	T1				
MVPA/weekday (mins)	72.3	23.8	78.8	31.2	8.6 [2.8 to 14.5]
MVPA/weekday after school (mins)^†^	13.1	7.0	16.6	9.3	4.0 [1.6 to 6.4]
CPM/weekday	653.6	176.2	680.4	224.7	46.7 [1.5 to 91.9]
CPM/weekday after school^†^	707.9	352.1	774.9	365.2	68.7 [−28.7 to 166.1]
	T2				
MVPA/weekday (mins)	63.1	26.5	64.3	22.3	−0.59 [−8.5 to 7.3]
MVPA/weekday after school (mins)^†^	12.5	8.5	12.0	8.0	−0.78 [−3.1 to 1.5]
CPM/weekday	527.3	171.8	546.1	150.9	7.5 [−50.4 to 65.5]
CPM/weekday after school^†^	559.6	286.8	566.0	274.8	4.3 [−96.7 to 105.3]

T0: Baseline; T1: T0 + 20 weeks; T2: T0 + 6 months.

**Between group differences always compare to the intervention arm and are adjusted for baseline outcome value, IMD, school size, percentage of girls recruited, percentage of Y5 pupils recruited, LEA, and school-level clustering

^†^After school period =3.30 to 8.30 pm.

^††^I = intervention; C = control.

**Table 7:** **Physical activity data by trial arm at T0, T1, and T2 and adjusted between group differences at T1 and T2 for girls with at least two days of valid weekday data for T0, T1, and T2 (n = 184)**

	**Control (n = 157)**		**Intervention (n = 153)**		
	**Mean**	**SD**	**Mean**	**SD**	**I vs. C** ^**††**^ adjusted difference in means (95% CI)**
	T0				
MVPA/weekday (mins)	53.7	16.3	51.7	15.0	N/A
MVPA/weekday after school (mins)^†^	11.7	5.4	11.3	6.1	N/A
CPM/weekday	501.5	118.0	498.8	111.9	N/A
CPM/weekday after school^†^	581.8	220.5	592.4	276.1	N/A
	Time 1				
MVPA/weekday (mins)	60.1	18.0	58.2	21.6	0.15 [−9.7 to 10.0]
MVPA/weekday after school (mins)^†^	12.9	7.0	13.0	7.6	−0.01 [−2.2 to 2.2]
CPM/weekday	583.3	171.1	577.9	184.6	16.0 [−73.1 to 105.0]
CPM/weekday after school^†^	707.6	405.1	720.7	421.6	41.6 [−104.8 to 188.0]
	Time 2				
MVPA/weekday (mins)	49.2	16.9	49.1	18.2	0.24 [−5.3 to 5.8]
MVPA/weekday after school (mins)^†^	11.1	6.4	10.4	6.5	−0.60 [−2.2 to 1.1]
CPM/weekday	440.5	116.4	446.6	120.6	−2.6 [−36.2 to 30.9]
CPM/weekday after school^†^	533.8	243.9	524.7	288.7	−17.2 [−81.8 to 47.3]

T0: Baseline; T1: T0 + 20 weeks; T2: T0 + 6 months.

*Between group differences always compare to the intervention arm and are adjusted for school-level clustering.

**Between group differences always compare to the intervention arm and are adjusted for baseline outcome value, IMD, school size, percentage of girls recruited, percentage of Y5 pupils recruited, LEA, and school-level clustering.

^†^After school period =3.30 to 8.30 pm.

^††^I = intervention; C = control.

A sample size calculation for a future trial focusing on all children and repeated to provide adequate power for all analyses to be stratified by sex is shown in Table 8. Using an alpha of 5% and power of 80% and based on the upper 95% limit of the CI of the ICC found in this feasibility study, a power calculation allowing for attrition of 20% as well as school-level clustering suggests that a full trial would require 900 pupils sampled from 15 schools per arm to detect a 10 minute difference in weekday MVPA. Analysis stratified by sex would increase this to 1020 pupils sampled from 17 schools per arm (30 children per school).

**Table 8:** **Sample size calculations based on detecting a 10 minute difference in weekday MVPA using a cluster size of 24 and 12 per school**

**Outcome** ^**a**^	**Cluster size**	**ICC** ^**b**^	**α**	**Power (β)**	**n required per arm** ^**c**^	**n schools/arm**	**N per arm inflated for attrition** ^**d**^	**Total N** ^**e**^
Cluster size = 24								
MVPA/weekday (min)	24	0.12977	5%	80%	360	15	450	900
MVPA/weekday (min)	24	0.12977	5%	90%	456	19	570	1140
MVPA/weekday (min)	24	0.12977	1%	90%	624	26	780	1560
		ICC^f^						
MVPA/weekday (min)	24	0.06534	5%	80%	240	10	300	600
MVPA/weekday (min)	24	0.06534	5%	90%	312	13	390	780
MVPA/weekday (min)	24	0.06534	1%	90%	408	17	510	1020
Cluster size = 12								
	**Cluster size**	**ICC** ^**b**^	**α**	**Power (β)**	**n required per arm** ^**c**^	**n schools/arm**	**N per arm inflated for attrition** ^**d**^	**Total N** ^**g**^
MVPA/weekday (min)	12	0.12977	5%	80%	204	17	255	510
MVPA/weekday (min)	12	0.12977	5%	90%	276	23	345	690
MVPA/weekday (min)	12	0.12977	1%	90%	384	32	480	960
		ICC^f^						
MVPA/weekday (min)	12	0.06534	5%	80%	156	13	195	390
MVPA/weekday (min)	12	0.06534	5%	90%	204	17	255	510
MVPA/weekday (min)	12	0.06534	1%	90%	276	23	345	690

^a^Target difference = 10 minutes of MVPA per weekday.

^b^observed ICC (95% CI) was 0.06534 (0.00091 to 0.12977); upper limit of 95% CI used.

^c^cluster size of 24.

^d^estimated attrition = 20% (~6 per cluster).

^e^based on 30 pupils per school (i.e. 24 + 6).

^f^Point estimate of ICC used.

^g^based on 15 pupils per school (i.e. 12 + 3).

The resource information shown in Table 9 indicates that over 50% of total indicative costs are non-recurrent, developmental and one-off training costs for teaching assistants, suggesting that the financial cost of running Action 3.30 falls substantially after the first year. There is some underlying variation in the hourly costs claimed by teaching assistants for attending training and delivery of Action 3.30 that is not apparent from estimation at school-level. However, using actual costs claimed as the basis for costing is likely to be closer to ‘real world’ delivery than costing on the basis that all 20 sessions were delivered twice a week by two teaching assistants in 10 schools.

**Table 9:** **Description of resources, mean (SD) unit costs £, units, indicative total cost 2012–13 prices**

**Category and description of resources used**	***Mean (SD) unit cost £***	***Number of units***	***Total cost £***
**Non-recurrent resources-development**			
**Leader consultation and development work**			
Refinement programme by Bristol City Council	200/day	6 days	1,200
Refinement programme after external input	200/day	3 days	600
Drafting 40 physical activity plans	200/day	15 days	3,000
**One-off resources- training**			3,800
Leader training TAs in groups	200/day	5 days/TA	300
First Aid training for TAs			
TA training/school (2 TAs/school*25 hours)^a^	494(83)/school	10 schools	4,940
TA session plan overview	60(56)/school	10 schools	600
**Recurrent resources- preparation**			
Printing training manuals	14/manual	20 manuals	280
Printing leader’s manual for TAs	18/manual	20 manuals	360
**Recurrent resources- Action 3.30 delivery**			
Booster training sessions for TAs	36(51)/school	10 schools	360
Intervention delivery by TAs up to 40 sessions^b^	858(173)/school	10 schools	8,620
Leader intervention delivery			
1 visit per school Bristol by train	7.20/school	5 schools	36
1 visit per school Bath by train	14.40/school	5 schools	72
Delivery of booster session	200/day	3 days	600
Room hire booster session			200
Email/phone support	25/hour	3 hours/10 schools	750
**Printing materials for Action 3.30 delivery** ^**c**^			1,155
**School sports equipment** ^**d**^			2,177
Indicative total cost			29,050
Indicative total cost excluding non-recurrent costs			24,250
Indicative total excluding one-off training and non-			
recurrent costs			14,610
Indicative cost per school (first year of delivery)			2,425
Indicative cost per pupil (first year of delivery) (n = 30)			81
Indicative cost per school (mainstream delivery)			1,461
Indicative cost per pupil (n = 30)			49

^a^5 day training programme 25 hours per school, session planning overview per school.

^b^Two TAs per school, TA could claim for up to 80 hours, hourly wage rate varies from £8.32 to £15, hours claimed range (0-80 hrs), average hours claimed 73.9 hours, based on 30 pupils per school.

^c^Club pledge, pupil pledge, parental feedback, certificates, Easter reminder flyers, Easter parent flyers.

^d^Branded sports equipment, sponge size 5 balls, frisbees, balloons, hockey sticks.

## Discussion

The data presented in this paper demonstrate that after-school physical activity programmes can be delivered by teaching assistants, and that it is feasible to deliver the Action 3:30 intervention in state funded primary schools. Nine out of ten schools delivered at least 38 of the 40 planned sessions. The data also show that it is possible to recruit participants to the study with around a third of eligible children consenting to join. However, as the children who signed up engaged in 58 minutes of daily MVPA at baseline, it could be argued that the programme appealed to an already active group of children rather than attracting children who would gain more benefit from participating in a physical activity programme. As such, alternative approaches might be needed to encourage less active children to join the club. The average weekly attendance levels were also variable, ranging from 36% to 86%, suggesting that strategies need to be found to maintain attendance and interest. The overall intervention had evidence of promise for an effect on weekday MVPA with 4.3 more minutes of MVPA in the intervention than the control group at the first follow-up assessment. Further examination indicated that results were not uniform for boys and girls. All effects were attenuated to the null when the club stopped running. The results therefore suggest that although the provision of the club created an opportunity to be physically active, increases in levels of activity ceased once the club sessions finished. As such, maintaining the increases attributable to participation in club activities will be difficult when clubs stop running and the most likely means of maintaining activity levels is to continue to run the sessions. The continued provision of the clubs would be consistent with normal school provision where a range of activities are provided after-school [13] and is therefore likely to be sustainable. Thus, the Action 3:30 intervention shows potential as an intervention approach but further refinements are needed to maximise effectiveness.

A 2012 meta-analysis of physical activity interventions for children that employed objective measures reported that the average effect of interventions was 4 additional minutes of MVPA per day [32]. The overall effect in this study (both boys and girls) was 4.3 minutes of weekday MVPA at T1, which is comparable to the average effect of previous physical activity interventions. However, for boys only, the effect was 8.6 minutes of MVPA, which is double the average effect of earlier interventions. If a similar effect could be extended to girls, Action 3:30 could make an important contribution to helping children to be physically active. It is important to note, however, that there was no evidence of an effect at the T2 follow-up, suggesting that any impact of the intervention is limited to improvements in habitual MVPA whilst the club is still running.

A number of school-based interventions have reported that the effects of school-based physical activity interventions have differed for boys and girls. For example, in the M-SPAN study, a two year school-based intervention that targeted healthy eating and physical activity promotion in US middle schools, the intervention led to an increase in boys’ MVPA but not girls’ [33]. Similarly, Salmon and colleagues reported that in the three-arm Switch-play project, in which participants received either a behaviour modification programme, a fundamental movement skills programme or a combination of both, sex was a moderator of the effect on vigorous-intensity physical activity [34]. Finally, Magnusson and colleagues reported that a multi-component physical activity intervention delivered by trained teachers to Icelandic school children, had a more favourable impact on MVPA in boys than girls [35]. Collectively, these findings indicate a need for further work to understand why school-based physical activity interventions are less successful for girls than boys (in terms of increasing MVPA levels) and highlight a requirement to enhance intervention effectiveness in girls.

### Feasibility of the intervention approach in relation to costs

It is common in most UK primary schools for external providers to be paid for delivering extra-curricular physical activity sessions [13] which creates an expectation that any existing school staff member who delivers physical activity sessions outside of their normal duties will also be paid. As such a key component of this study was to identify the potential costs of delivering the Action 3:30 intervention. The indicative average cost of this intervention was £2,425 per school or £81 per participating child during its first year and £1,461 per school or £49 per participating child thereafter. Comparing these costs to previous studies is difficult as there are very few studies that have reported the cost of delivering children’s physical activity interventions. However, we recently reported that the average cost of a 10-week after-school dance intervention for Year 7 girls was £1,329 per school based on 30 girls per school [36]. In a US study, Wang and colleagues reported that the delivery costs of the Fit Kid Project, in which an after-school programme including 40 minutes of academic enrichment, a healthy snack and 80 minutes of MVPA a day for 5 days a week during the whole school year, was $558 per child. The programme was delivered in 18 elementary schools in Augusta, Georgia with the prices based on 2003 costs [37]. When compared with our earlier dance project (delivered over 10 weeks) and the more intense Fit Kid project, Action 3:30 appears to offer an economically viable intervention. This would need to be confirmed, however, via a full trial analysing resource use and prices separately during cost estimation and taking full account of variations in costs between schools.

The information in Table 8 shows that a study powered to detect a 10 minute increase in MVPA would require between 10 and 24 schools with a sample of 24 pupils per school for analysis. Moreover, if a future trial were to be designed to analyse differences within each sex the overall sample size would need to be between 13 and 32 schools per arm, with an average of 12 children per sex, per school included in the analysis. Such a trial size would be comparable to previous school-based interventions. For example, the current Active for Life Year 5 (AFLY5) project - focussing on increasing physical activity and fruit and vegetable consumption - is currently running in 60 UK primary schools [38]. Similarly, the Healthy Lifestyles Programme (HELP), which is testing whether a drama based programme can reduce obesity and increase physical activity, is currently being evaluated in 32 primary schools [39].

### Strengths and limitations

The major strength of this study is the careful development, and robust feasibility trial evaluation, of the Action 3:30 intervention via a randomised controlled trial design conducted in line with guidance on the development and evaluation of complex interventions [40]. In conducting this evaluation we have gathered the information required to refine the intervention and design a definitive trial. An inherent limitation in research at this stage of development is that the study was not powered to detect differences between the intervention and control groups and it is therefore not possible to draw firm conclusions about the effectiveness of the Action 3:30 school-based intervention based on the information presented here. The study is also limited by the lack of information on the extent to which the teaching assistants adopted autonomy supportive teaching styles. A recent study [41] used direct observation of physical education teachers’ teaching style to assess the extent to which the teachers adopted an autonomy supportive teaching style. Such a tool could be adapted for use with teaching assistants and utilised in a future trial of the intervention. It is also important to acknowledge that a higher proportion of intervention participants were excluded from the final analyses due to incomplete accelerometer data and that, to maximise the sample size, a two-weekday accelerometer inclusion criteria was used for the analyses. Although there were no differences in the BMI, IMD or baseline MVPA of those with two versus three days of accelerometer data there was some evidence that excluded participants were older and from more deprived households. As such, strategies to enhance levels of data provision (particularly the provision of adequate accelerometer data) are likely to be required before proceeding to a larger trial. Thus, data presented in this paper suggest that refinements to the intervention content will be required before proceeding to a definite trial. Further work that explores these issues, such as how to increase choice and increase the interest of the girls in the study, will be explored in a separate paper which explores qualitative data from interviews with teaching assistants and school contacts, and focus groups with the children.

## Conclusions

The data presented in this article demonstrate that it is feasible to engage teaching assistants in training to deliver after-school physical activity programmes and that the Action 3:30 intervention is an programme that could hold considerable promise if some adaptations were made to the content. When examined for the entire sample the impact of the Action 3:30 intervention was comparable to previous physical activity interventions, but further analysis indicated that there was a marked sex difference with a positive impact on boys and no evidence of an effect on girls. The Action 3:30 intervention holds considerable promise as an approach to increase physical activity among primary school aged children but further refinements are needed to enhance the effectiveness of the intervention, particularly for girls.

### Data sharing

Anonymised versions of the data from the Action 3:30 project have been deposited in the University of Bristol Research Data Repository (http://data.bris.ac.uk/data/) and will be made available to external collaborators from September 2016. Links to the data and papers will be made available on the study website (http://www.bristol.ac.uk/sps/research/researchprojectpages/action330/index.html).
